# Supplementary material for: Occupational Factors and Socioeconomic Differences in Breast Cancer Risk and Stage at Diagnosis in Swiss Working Women
Source: Cancers (Basel). 2022 Jul 29;14(15):3713. doi: 10.3390/cancers14153713 (PMC9367372; doi:10.3390/cancers14153713)
Supplement: Supplementary file 1 [file cancers-14-03713-s001.zip › Figures S3-S4.pdf]

(a)

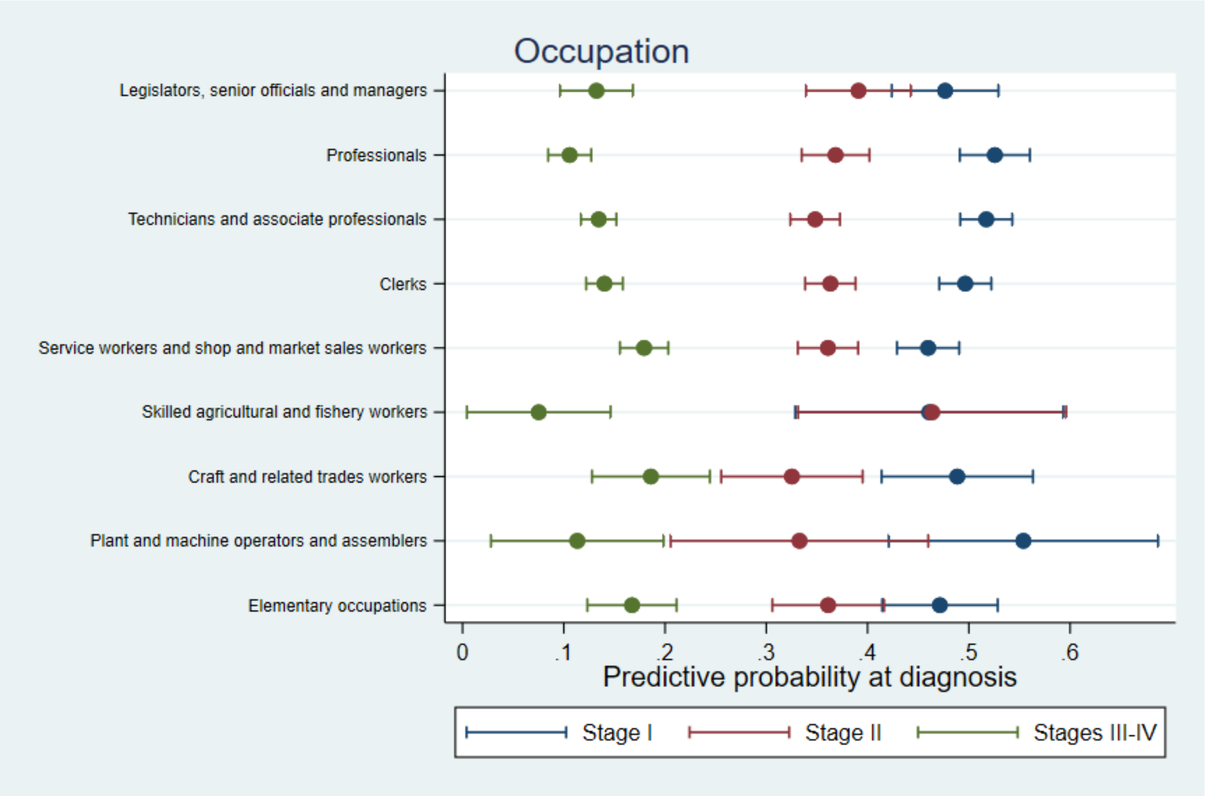
Occupation
Legislators, senior officials and managers
Professionals
Technicians and associate professionals
Clerks
Service workers and shop and market sales workers
Skilled agricultural and fishery workers
Craft and related trades workers
Plant and machine operators and assemblers
Elementary occupations
0
.1
.2
.3
.4
.5
.6
Predictive probability at diagnosis
Stage I
Stage II
Stages III-IV

(b)

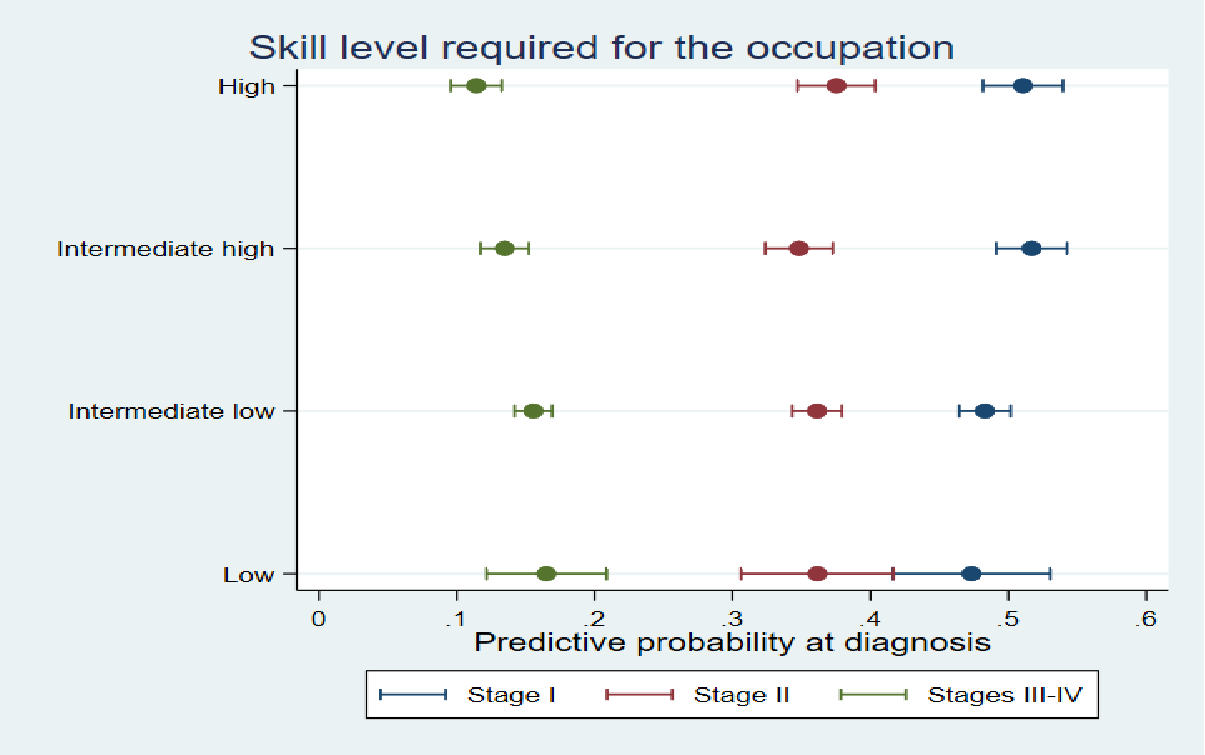
Skill level required for the occupation
High
Intermediate high
Intermediate low
Low
0
.1
.2
.3
.4
.5
.6
Predictive probability at diagnosis
Stage I
Stage II
Stages III-IV

(c)

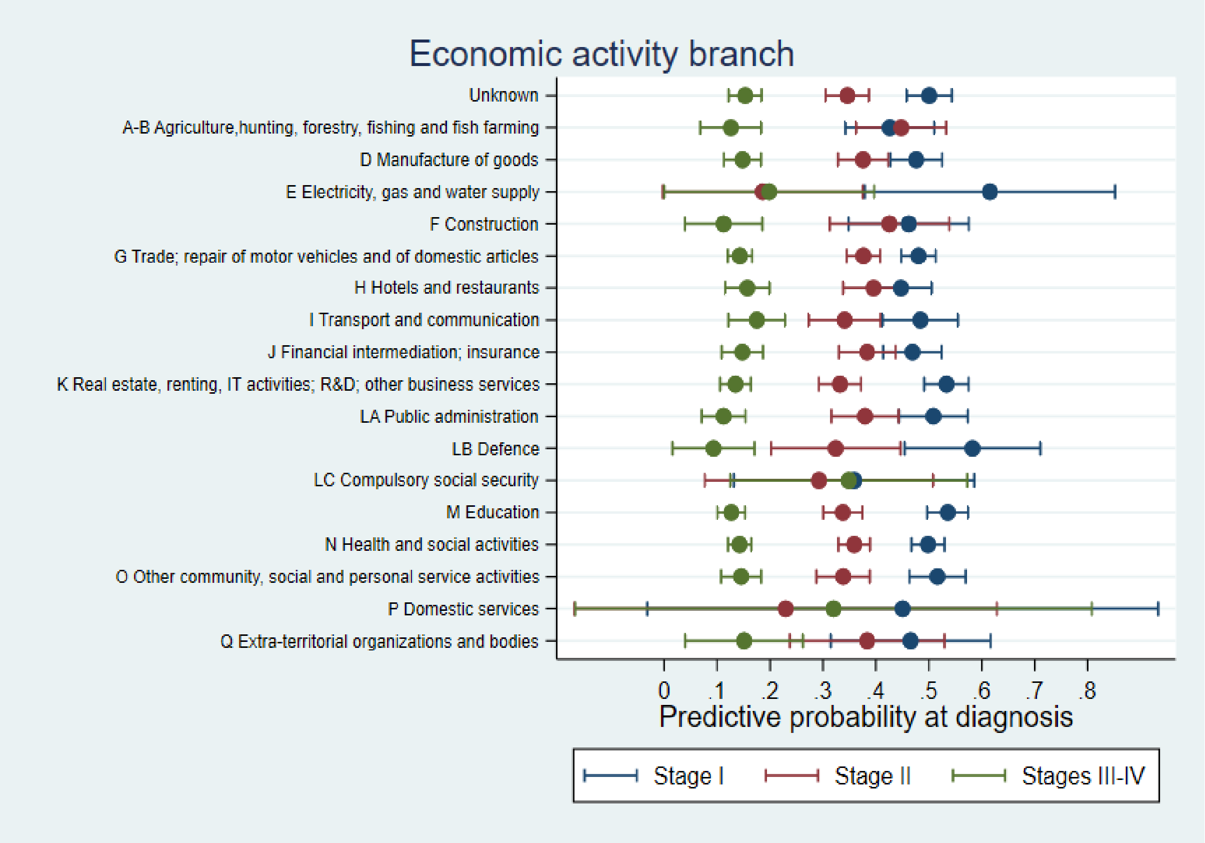

(d)

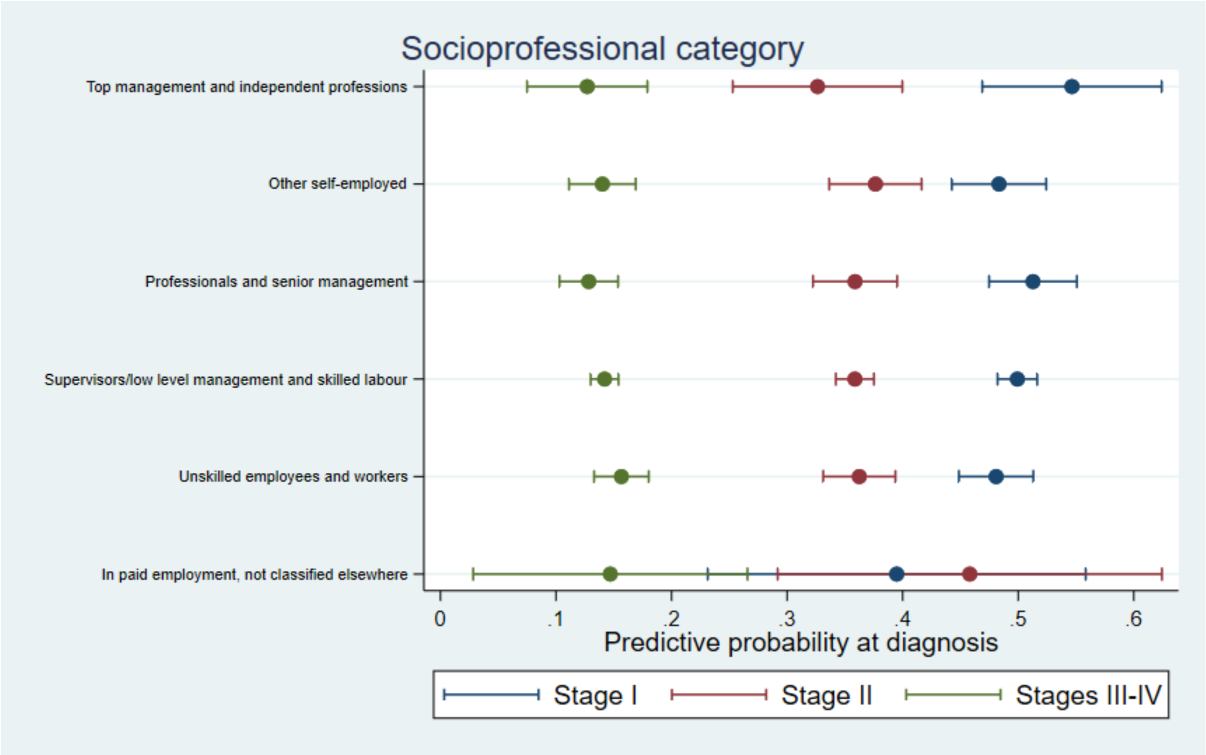

Figure S3. Predictive probability of being diagnosed with breast cancer of stage I, II or III-IV by (a) occupation, (b) skill level required for the occupation, (c) economic activity branch and (d) socioprofessional category, adjusted for age, calendar time, canton, marital status and nationality, females in western Switzerland, 2000-2014

(a)

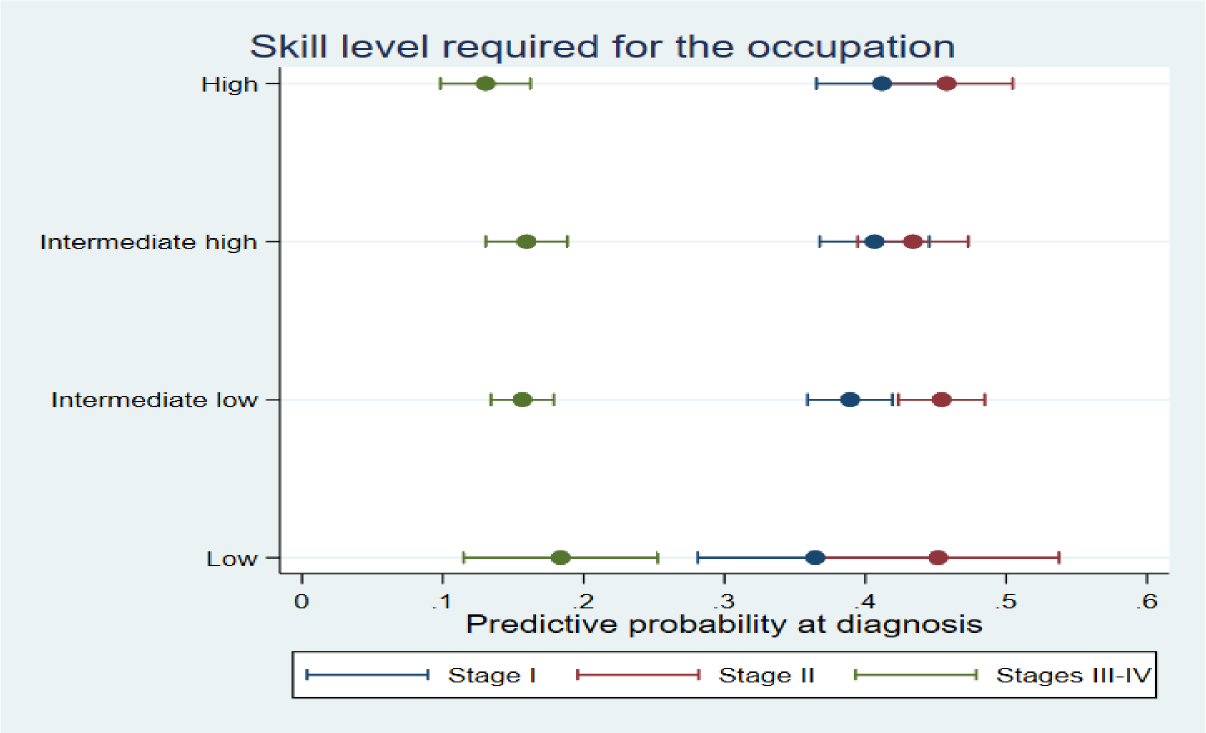
Skill level required for the occupation
High
Intermediate high
Intermediate low
Low
0
.1
.2
.3
.4
.5
.6
Predictive probability at diagnosis
Stage I
Stage II
Stages III-IV

(b)

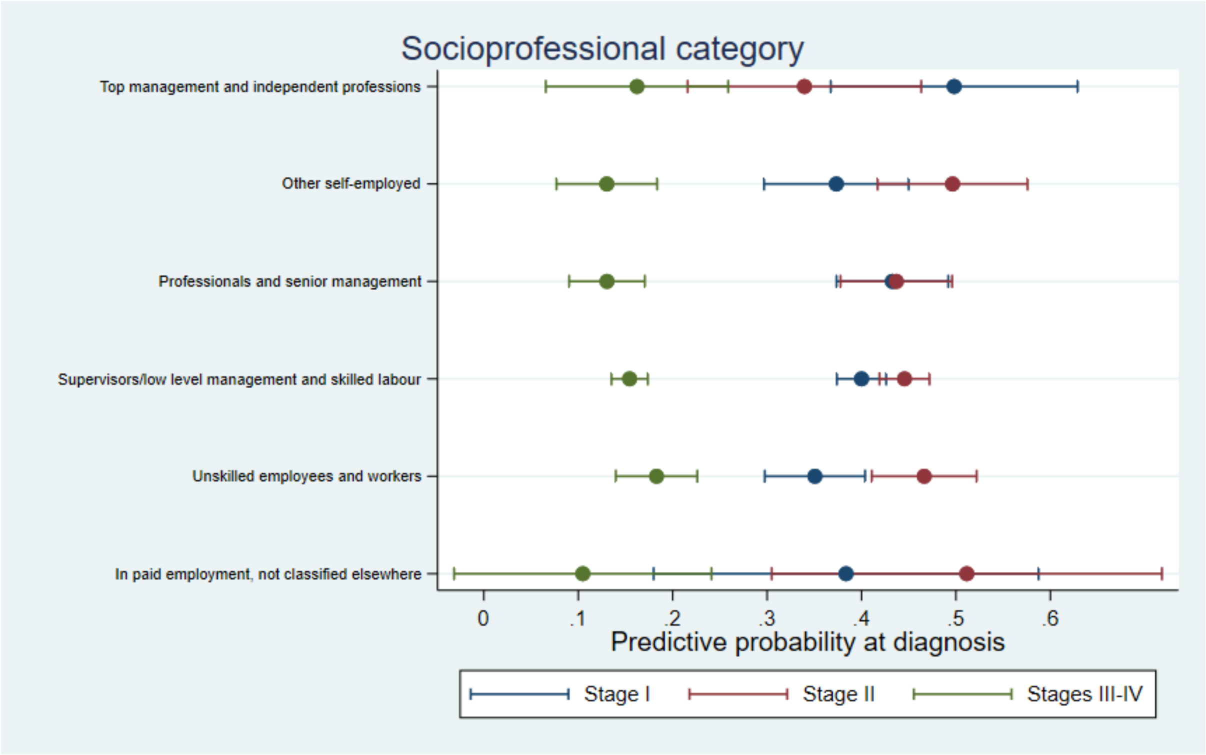
Socioprofessional category
Top management and independent professions
Other self-employed
Professionals and senior management
Supervisors/low level management and skilled labour
Unskilled employees and workers
In paid employment, not classified elsewhere
0
.1
.2
.3
.4
.5
.6
Predictive probability at diagnosis
Stage I
Stage II
Stages III-IV

(c)

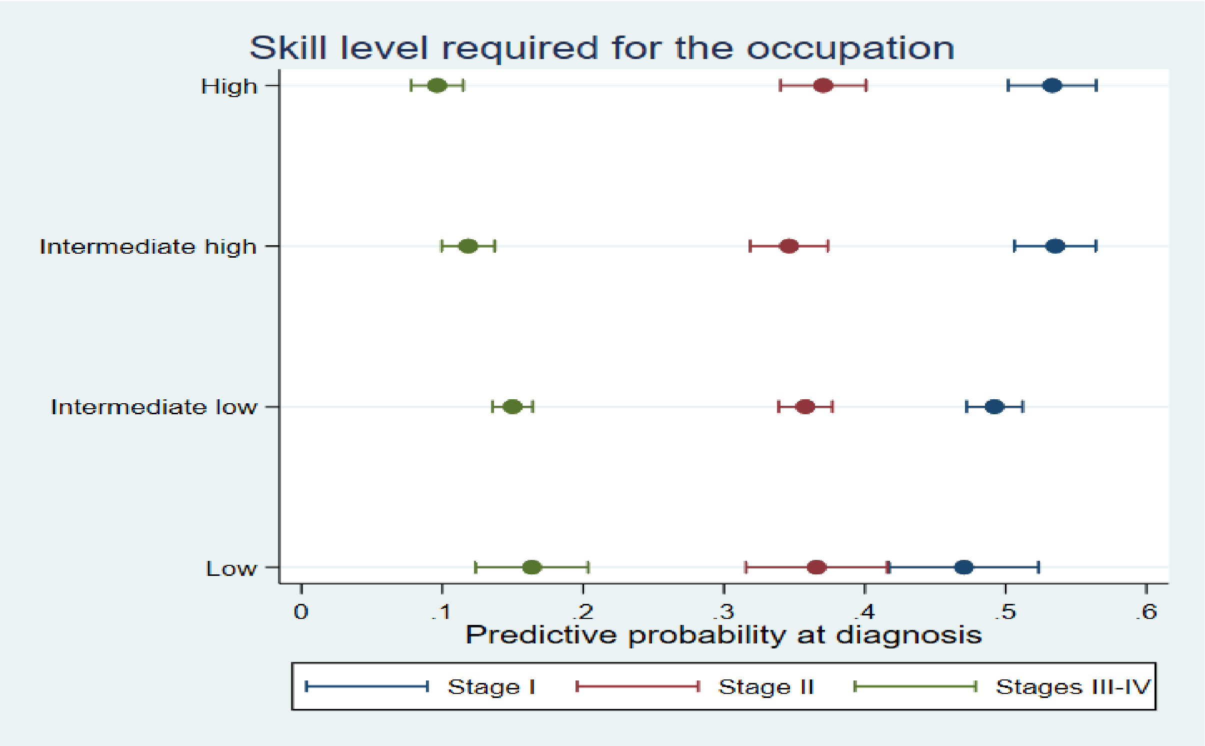

(d)

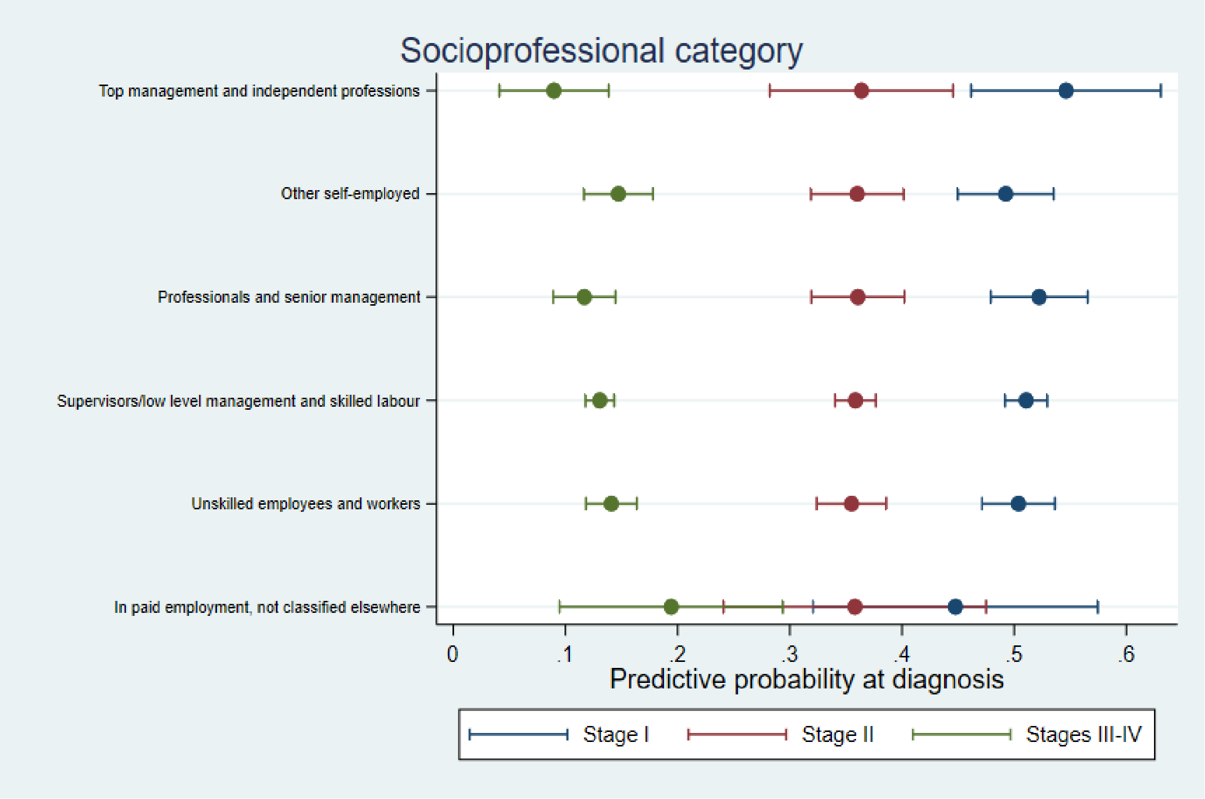

Figure S4. Predictive probability of being diagnosed with breast cancer of stage I, II or III-IV for women below age 50 by (a) skill level required for the occupation and (b) socioprofessional category, and for women aged 50 and over by (c) skill level required for the occupation and (d) socioprofessional category, adjusted for age, calendar time, canton, marital status and nationality, females in western Switzerland, 1990-2014
