# Supplementary material for: Occupational Factors and Socioeconomic Differences in Breast Cancer Risk and Stage at Diagnosis in Swiss Working Women
Source: Cancers (Basel). 2022 Jul 29;14(15):3713. doi: 10.3390/cancers14153713 (PMC9367372; doi:10.3390/cancers14153713)
Supplement: Supplementary file 1 [file cancers-14-03713-s001.zip › Tables_S1-S3rev.pdf]

**Table S1**: Relative risk (RR) with confidence interval (95%CI) of breast cancer by occupational, socio-professional, economical activity and skill level category, among females aged 18-85 years in western Switzerland, 2005-2014

| **Occupational variables** | **Nb cases** | **Person-years (in 100'000)** | **Models 1* RR** | | **Models 2 ** RR** | | **Models 3 *** RR** | |
|---|---|---|---|---|---|---|---|---|
| ***Occupation[a]*** | | | **p<0.001** | | **p<0.001** | | **p<0.001** | |
| 1. Legislators, senior officials and managers | 259 | 1.17 | 1.00 | Ref. | 1.00 | Ref. | 1.00 | Ref. |
| 2. Professionals | 630 | 2.64 | 1.08 | [0.93,1.24] | 1.14 | [0.99,1.32] | 1.15 | [0.99,1.33] |
| 3. Technicians and associate professionals | 1'176 | 5.96 | 0.89 | [0.77,1.02] | 0.97 | [0.85,1.11] | 0.98 | [0.85,1.12] |
| 4. Clerks | 1'176 | 5.50 | 0.96 | [0.84,1.10] | 1.05 | [0.91,1.20] | 1.05 | [0.92,1.20] |
| 5. Service workers and shop and market sales workers | 868 | 4.69 | **0.83** | **[0.72,0.96]** | 0.91 | [0.79,1.05] | 0.91 | [0.79,1.05] |
| 6. Skilled agricultural and fishery workers | 47 | 0.29 | 0.73 | [0.53,1.00] | 0.78 | [0.57,1.06] | 0.78 | [0.57,1.06] |
| 7. Craft and related trades workers | 133 | 0.68 | 0.89 | [0.72,1.09] | 0.97 | [0.79,1.20] | 0.98 | [0.79,1.21] |
| 8. Plant and machine operators and assemblers | 44 | 0.24 | 0.81 | [0.58,1.12] | 0.81 | [0.59,1.11] | 0.81 | [0.58,1.11] |
| 9. Elementary occupations | 225 | 1.10 | 0.92 | [0.77,1.10] | 0.89 | [0.74,1.06] | 0.88 | [0.74,1.06] |
| ***Socioprofessional category*** | | | **p<0.001** | | **p<0.001** | | **p=0.003** | |
| Top management and independent professions | 130 | 0.50 | 1.00 | Ref. | 1.00 | Ref. | 1.00 | Ref. |
| Other self-employed | 467 | 1.66 | 1.07 | [0.88,1.31] | 1.04 | [0.86,1.27] | 1.03 | [0.85,1.26] |
| Professionals and senior management | 504 | 2.21 | 0.87 | [0.71,1.05] | 1.00 | [0.83,1.21] | 1.01 | [0.83,1.23] |
| Supervisors/low level management and skilled labour | 2'608 | 13.80 | **0.72** | **[0.60,0.86]** | 0.88 | [0.74,1.05] | 0.89 | [0.74,1.06] |
| Unskilled employees and workers | 830 | 4.01 | **0.79** | **[0.65,0.95]** | 0.85 | [0.71,1.02] | 0.86 | [0.71,1.04] |
| In paid employment, not classified elsewhere | 19 | 0.09 | 0.81 | [0.50,1.32] | 0.90 | [0.56,1.46] | 0.90 | [0.56,1.46] |
| ***Skill level required for the occupation*** | | | **p<0.001** | | **p=0.006** | | **p=0.007** | |
| Lowest skill level | 225 | 1.10 | 1.00 | Ref. | 1.00 | Ref. | 1.00 | Ref. |
| 2nd lowest skill level | 2'268 | 11.41 | 0.98 | [0.85,1.12] | 1.10 | [0.96,1.26] | 1.10 | [0.96,1.26] |
| 2nd highest skill level | 1'176 | 5.96 | 0.97 | [0.84,1.12] | 1.10 | [0.95,1.27] | 1.10 | [0.95,1.27] |
| Highest skill level | 889 | 3.81 | 1.15 | [0.99,1.33] | **1.23** | **[1.07,1.43]** | **1.23** | **[1.06,1.43]** |
| ***Economic activity branch[b]*** | | | **p<0.001** | | p=0.059 | | p=0.123 | |
| Unknown | 424 | 1.88 | 1.07 | [0.95,1.21] | 1.04 | [0.92,1.17] | 1.04 | [0.92,1.17] |
| A-B Agriculture,hunting, forestry, fishing and fish farming | 119 | 0.48 | 1.18 | [0.97,1.43] | 1.11 | [0.91,1.34] | 1.10 | [0.90,1.33] |
| C Mining and quarring | 2 | 0.01 | 1.26 | [0.31,5.18] | 1.23 | [0.31,4.92] | 1.24 | [0.31,4.98] |
| D Manufacture of goods | 310 | 1.65 | 0.89 | [0.78,1.02] | 0.96 | [0.84,1.09] | 0.97 | [0.85,1.10] |
| E Electricity, gas and water supply | 12 | 0.07 | 0.82 | [0.46,1.46] | 0.87 | [0.49,1.53] | 0.87 | [0.49,1.54] |

| | | | | | | | | |
|---|---|---|---|---|---|---|---|---|
| F Construction | 69 | 0.34 | 0.96 | [0.75,1.23] | 0.96 | [0.75,1.23] | 0.96 | [0.75,1.22] |
| G Trade; repair of motor vehicles and of domestic articles | 710 | 3.73 | 0.91 | [0.82,1.00] | 0.95 | [0.86,1.05] | 0.96 | [0.86,1.06] |
| H Hotels and restaurants | 233 | 1.29 | 0.86 | [0.74,1.00] | 0.95 | [0.82,1.09] | 0.96 | [0.83,1.11] |
| I Transport and communication | 161 | 0.96 | **0.80** | **[0.67,0.94]** | 0.89 | [0.75,1.05] | 0.90 | [0.76,1.06] |
| J Financial intermediation; insurance | 244 | 1.32 | 0.88 | [0.76,1.02] | 0.98 | [0.85,1.14] | 0.99 | [0.86,1.15] |
| K Real estate, renting, IT activities; research and development; other business services | 442 | 2.15 | 0.98 | [0.87,1.10] | 1.05 | [0.94,1.18] | 1.07 | [0.95,1.20] |
| LA Public administration | 192 | 0.72 | **1.28** | **[1.09,1.50]** | **1.23** | **[1.05,1.45]** | **1.22** | **[1.04,1.43]** |
| LB Defence | 47 | 0.25 | 0.90 | [0.67,1.22] | 0.94 | [0.70,1.27] | 0.94 | [0.70,1.26] |
| LC Compulsory social security | 16 | 0.07 | 1.14 | [0.69,1.88] | 1.19 | [0.73,1.96] | 1.20 | [0.73,1.96] |
| M Education | 498 | 2.10 | **1.13** | **[1.01,1.27]** | 1.06 | [0.95,1.19] | 1.06 | [0.95,1.19] |
| N Health and social activities | 792 | 3.76 | 1.00 | Ref. | 1.00 | Ref. | 1.00 | Ref. |
| O Other community, social and personal service activities | 267 | 1.38 | 0.92 | [0.80,1.06] | 0.94 | [0.82,1.08] | 0.95 | [0.83,1.09] |
| Q Extra-territorial organizations and bodies | 20 | 0.13 | 0.71 | [0.45,1.11] | **0.58** | **[0.37,0.90]** | **0.60** | **[0.39,0.94]** |

[a] Occupation is coded on 1 digit using the International Classification of Occupations, version 1988 (ISCO-88).

[b] Economic activity/industry is coded using the General Classification of Economic Activities (NOGA), based on ISCI third and NACE first revisions

* Univariate model

** Adjusted for age, period and canton

*** Adjusted for age, period, canton, civil status, civil status x age and nationality

Statistically significant estimates and p-values<0.05 are shown in bold

**Table S2**: Relative risk (RR) with confidence interval (95%CI) of breast cancer by occupational, socio-professional, economical activity and skill level category, among females aged 18-49 years in in Swiss cantons of Neuchâtel, Geneva, Vaud and Wallis, 1990-2014

| **Occupational variables** | **Nb cases** | **Person-years (in 100'000)** | **Models 1* RR** | | **Models 2 ** RR** | | **Models 3 *** RR** | |
|---|---|---|---|---|---|---|---|---|
| ***Occupation[a]*** | | | **p<0.001** | | **p=0.002** | | **p<0.001** | |
| 1. Legislators, senior officials and managers | 143 | 1.61 | 1.00 | Ref. | 1.00 | Ref. | 1.00 | Ref. |
| 2. Professionals | 415 | 3.97 | 1.19 | [0.96,1.47] | 1.18 | [0.95,1.46] | 1.22 | [0.99,1.52] |
| 3. Technicians and associate professionals | 757 | 9.01 | 1.06 | [0.86,1.29] | 1.07 | [0.88,1.31] | 1.11 | [0.90,1.35] |
| 4. Clerks | 704 | 9.43 | 1.00 | [0.82,1.23] | 1.03 | [0.84,1.26] | 1.04 | [0.85,1.28] |
| 5. Service workers and shop and market sales workers | 469 | 7.14 | 0.86 | [0.69,1.05] | 0.89 | [0.72,1.10] | 0.89 | [0.72,1.10] |
| 6. Skilled agricultural and fishery workers | 28 | 0.43 | 0.77 | [0.49,1.21] | 0.79 | [0.50,1.25] | 0.78 | [0.49,1.24] |
| 7. Craft and related trades workers | 93 | 1.07 | 0.98 | [0.73,1.30] | 0.98 | [0.73,1.31] | 1.00 | [0.74,1.33] |
| 8. Plant and machine operators and assemblers | 23 | 0.36 | 0.69 | [0.43,1.11] | 0.68 | [0.42,1.10] | 0.68 | [0.42,1.09] |
| 9. Elementary occupations | 211 | 3.68 | 0.87 | [0.68,1.11] | 0.83 | [0.65,1.07] | 0.80 | [0.62,1.02] |
| ***Socioprofessional category*** | | | **p<0.001** | | **p<0.001** | | **p<0.001** | |
| Top management and independent professions | 73 | 0.53 | 1.00 | Ref. | 1.00 | Ref. | 1.00 | Ref. |
| Other self-employed | 195 | 1.81 | 0.83 | [0.62,1.13] | 0.87 | [0.64,1.18] | 0.81 | [0.59,1.09] |
| Professionals and senior management | 332 | 3.16 | 0.78 | [0.59,1.04] | 0.77 | [0.58,1.02] | 0.80 | [0.60,1.07] |
| Supervisors/low level management and skilled labour | 1732 | 23.00 | **0.64** | **[0.50,0.83]** | **0.66** | **[0.51,0.86]** | **0.68** | **[0.53,0.89]** |
| Unskilled employees and workers | 477 | 7.30 | **0.58** | **[0.44,0.76]** | **0.58** | **[0.44,0.76]** | **0.59** | **[0.44,0.78]** |
| In paid employment, not classified elsewhere | 34 | 0.90 | **0.49** | **[0.30,0.79]** | **0.44** | **[0.27,0.72]** | **0.46** | **[0.28,0.75]** |
| ***Skill level required for the occupation*** | | | **p<0.001** | | **p=0.002** | | **p<0.001** | |
| Lowest skill level | 211 | 3.68 | 1.00 | Ref. | 1.00 | Ref. | 1.00 | Ref. |
| 2nd lowest skill level | 1317 | 18.44 | 1.07 | [0.90,1.27] | 1.15 | [0.96,1.37] | **1.20** | **[1.01,1.44]** |
| 2nd highest skill level | 757 | 9.01 | **1.22** | **[1.01,1.46]** | **1.29** | **[1.07,1.55]** | **1.37** | **[1.14,1.66]** |
| Highest skill level | 558 | 5.57 | **1.31** | **[1.08,1.58]** | **1.35** | **[1.11,1.63]** | **1.44** | **[1.18,1.74]** |
| ***Economic activity branch[b]*** | | | **p=0.002** | | **p=0.001** | | **p=0.009** | |
| Unknown | 169 | 1.98 | 0.94 | [0.77,1.14] | 0.95 | [0.78,1.15] | 0.95 | [0.78,1.15] |
| A-B Agriculture,hunting, forestry, fishing and fish farming | 53 | 0.55 | 1.01 | [0.73,1.39] | 1.04 | [0.75,1.45] | 0.96 | [0.70,1.33] |
| D Manufacture of goods | 241 | 3.29 | 0.86 | [0.72,1.03] | 0.85 | [0.70,1.01] | 0.85 | [0.71,1.03] |

| | | | | | | | | |
|---|---|---|---|---|---|---|---|---|
| E Electricity, gas and water supply | 6 | 0.10 | 0.54 | [0.23,1.28] | 0.53 | [0.22,1.26] | 0.54 | [0.23,1.28] |
| F Construction | 44 | 0.53 | 0.89 | [0.63,1.27] | 0.88 | [0.61,1.26] | 0.84 | [0.59,1.21] |
| G Trade; repair of motor vehicles and of domestic articles | 441 | 6.39 | **0.81** | **[0.70,0.94]** | **0.80** | **[0.69,0.93]** | **0.81** | **[0.69,0.94]** |
| H Hotels and restaurants | 155 | 2.38 | **0.79** | **[0.64,0.97]** | **0.79** | **[0.64,0.97]** | 0.81 | [0.66,1.00] |
| I Transport and communication | 96 | 1.71 | **0.63** | **[0.49,0.80]** | **0.60** | **[0.47,0.77]** | **0.62** | **[0.48,0.79]** |
| J Financial intermediation; insurance | 183 | 2.66 | 0.91 | [0.75,1.10] | 0.86 | [0.70,1.04] | 0.89 | [0.73,1.08] |
| K Real estate, renting, IT activities; research and development; other business services | 297 | 3.76 | 0.89 | [0.75,1.05] | 0.85 | [0.72,1.01] | 0.88 | [0.74,1.03] |
| LA Public administration | 115 | 1.20 | 1.06 | [0.83,1.34] | 1.01 | [0.79,1.28] | 0.98 | [0.77,1.25] |
| LB Defence | 18 | 0.20 | 0.87 | [0.53,1.42] | 0.90 | [0.55,1.49] | 0.90 | [0.55,1.48] |
| LC Compulsory social security | 5 | 0.06 | 0.79 | [0.31,1.99] | 0.79 | [0.31,2.00] | 0.80 | [0.32,2.02] |
| M Education | 320 | 3.23 | 1.17 | [0.99,1.38] | 1.12 | [0.95,1.33] | 1.12 | [0.94,1.32] |
| N Health and social activities | 484 | 5.91 | 1.00 | Ref. | 1.00 | Ref. | 1.00 | Ref. |
| O Other community, social and personal service activities | 182 | 2.20 | 0.90 | [0.74,1.09] | 0.87 | [0.72,1.06] | 0.89 | [0.73,1.08] |
| P Domestic services | 13 | 0.23 | 0.76 | [0.38,1.49] | 0.61 | [0.31,1.23] | 0.64 | [0.32,1.26] |
| Q Extra-territorial organizations and bodies | 20 | 0.30 | 0.83 | [0.50,1.37] | 0.68 | [0.40,1.14] | 0.75 | [0.45,1.26] |

[a] Occupation is coded using the International Classification of Occupations, version 1988 (ISCO-88), coded on 1 digit.

[b] Economic activity/industry is coded using the General Classification of Economic Activities (NOGA), based on ISCI third and NACE first revisions

* Univariate model

** Adjusted for age, time period and canton

*** Adjusted for age, time period, canton, civil status, civil status x age and nationality

Statistically significant estimates and p-values<0.05 are shown in bold

**Table S3**: Relative risk (RR) with confidence interval (95%CI) of breast cancer by occupational, socio-professional, economical activity and skill level category, among females aged 50-85 years in in Swiss cantons of Neuchâtel, Geneva, Vaud and Wallis, 1990-2014

| **Occupational variables** | **Nb cases** | **Person-years (in 100'000)** | **Models 1* RR** | | **Models 2 ** RR** | | **Models 3 *** RR** | |
|---|---|---|---|---|---|---|---|---|
| ***Occupation*[a]** | | | **p<0.001** | | **p=0.002** | | **p<0.001** | |
| 3. Technicians and associate professionals | 1367 | 4.36 | 0.96 | [0.85,1.09] | 0.98 | [0.86,1.11] | 0.97 | [0.86,1.10] |
| 4. Clerks | 1579 | 4.75 | 1.07 | [0.95,1.21] | 1.09 | [0.96,1.23] | 1.09 | [0.96,1.23] |
| 5. Service workers and shop and market sales workers | 1060 | 3.69 | 0.91 | [0.80,1.03] | 0.95 | [0.83,1.07] | 0.96 | [0.84,1.09] |
| 6. Skilled agricultural and fishery workers | 73 | 0.29 | 0.81 | [0.61,1.07] | 0.84 | [0.63,1.12] | 0.84 | [0.63,1.12] |
| 7. Craft and related trades workers | 182 | 0.638 | 0.87 | [0.72,1.05] | 0.87 | [0.71,1.05] | 0.88 | [0.72,1.06] |
| 8. Plant and machine operators and assemblers | 70 | 0.24 | 0.88 | [0.67,1.15] | 0.88 | [0.67,1.16] | 0.90 | [0.68,1.18] |
| 9. Elementary occupations | 496 | 2.12 | **0.78** | **[0.68,0.91]** | **0.76** | **[0.66,0.89]** | **0.78** | **[0.67,0.91]** |
| ***Socioprofessional category*** | | | **p<0.001** | | **p<0.001** | | **p<0.001** | |
| Top management and independent professions | 159 | 0.45 | 1.00 | Ref. | 1.00 | Ref. | 1.00 | Ref. |
| Other self-employed | 628 | 1.87 | 0.99 | [0.82,1.19] | 1.03 | [0.85,1.24] | 1.03 | [0.86,1.25] |
| Professionals and senior management | 594 | 1.67 | 1.04 | [0.86,1.25] | 1.04 | [0.86,1.25] | 1.04 | [0.86,1.25] |
| Supervisors/low level management and skilled labour | 3271 | 10.31 | 0.95 | [0.80,1.13] | 0.99 | [0.83,1.17] | 0.99 | [0.84,1.18] |
| Unskilled employees and workers | 1234 | 4.73 | **0.81** | **[0.68,0.96]** | **0.83** | **[0.70,0.99]** | 0.86 | [0.71,1.02] |
| In paid employment, not classified elsewhere | 89 | 0.35 | 0.91 | [0.67,1.22] | 0.86 | [0.64,1.17] | 0.87 | [0.65,1.18] |
| ***Skill level required for the occupation*** | | | **p<0.001** | | **p<0.001** | | **p<0.001** | |
| Lowest skill level | 496 | 2.12 | 1.00 | Ref. | 1.00 | Ref. | 1.00 | Ref. |
| 2nd lowest skill level | 2964 | 9.60 | **1.25** | **[1.12,1.39]** | **1.31** | **[1.17,1.46]** | **1.28** | **[1.15,1.43]** |
| 2nd highest skill level | 1367 | 4.36 | **1.23** | **[1.09,1.38]** | **1.27** | **[1.13,1.43]** | **1.23** | **[1.09,1.39]** |
| Highest skill level | 1148 | 3.30 | **1.37** | **[1.22,1.54]** | **1.39** | **[1.23,1.57]** | **1.34** | **[1.19,1.52]** |
| ***Economic activity branch*[b]** | | | **p=0.022** | | **p=0.050** | | p=0.067 | |
| Unknown | 467 | 1.37 | 1.10 | [0.98,1.24] | 1.09 | [0.97,1.23] | 1.10 | [0.98,1.24] |
| A-B Agriculture,hunting, forestry, fishing and fish farming | 132 | 0.48 | 0.91 | [0.74,1.11] | 0.93 | [0.76,1.15] | 0.93 | [0.76,1.15] |
| D Manufacture of goods | 491 | 1.70 | 0.99 | [0.88,1.12] | 0.98 | [0.87,1.11] | 0.99 | [0.87,1.12] |
| E Electricity, gas and water supply | 17 | 0.06 | 0.96 | [0.58,1.58] | 0.98 | [0.59,1.63] | 0.96 | [0.58,1.60] |
| F Construction | 80 | 0.31 | **0.77** | **[0.60,0.99]** | **0.76** | **[0.59,0.98]** | **0.76** | **[0.59,0.98]** |

| | | | | | | | | |
|---|---|---|---|---|---|---|---|---|
| G Trade; repair of motor vehicles and of domestic articles | 1064 | 3.45 | 1.03 | [0.93,1.14] | 1.02 | [0.92,1.13] | 1.03 | [0.93,1.14] |
| H Hotels and restaurants | 269 | 0.99 | 0.87 | [0.75,1.01] | 0.86 | [0.74,1.00] | 0.88 | [0.76,1.02] |
| I Transport and communication | 182 | 0.68 | 0.86 | [0.73,1.02] | 0.84 | [0.71,1.00] | 0.84 | [0.70,1.00] |
| J Financial intermediation; insurance | 335 | 1.03 | 1.10 | [0.96,1.27] | 1.05 | [0.91,1.21] | 1.04 | [0.90,1.20] |
| K Real estate, renting, IT activities; research and development; other business services | 532 | 1.63 | 1.06 | [0.94,1.19] | 1.02 | [0.90,1.15] | 1.02 | [0.91,1.15] |
| LA Public administration | 277 | 0.75 | **1.20** | **[1.03,1.39]** | 1.15 | [0.99,1.34] | 1.14 | [0.98,1.32] |
| LB Defence | 40 | 0.12 | 1.00 | [0.72,1.39] | 1.03 | [0.74,1.43] | 1.01 | [0.73,1.41] |
| LC Compulsory social security | 12 | 0.04 | 1.02 | [0.56,1.85] | 1.01 | [0.56,1.84] | 1.00 | [0.55,1.81] |
| M Education | 642 | 2.02 | 1.03 | [0.93,1.15] | 1.00 | [0.90,1.12] | 1.00 | [0.89,1.11] |
| N Health and social activities | 970 | 3.16 | 1.00 | Ref. | 1.00 | Ref. | 1.00 | Ref. |
| O Other community, social and personal service activities | 352 | 1.19 | 0.94 | [0.82,1.07] | 0.90 | [0.79,1.03] | 0.90 | [0.79,1.03] |
| P Domestic services | 28 | 0.14 | 0.80 | [0.51,1.23] | 0.70 | [0.45,1.10] | 0.73 | [0.47,1.13] |
| Q Extra-territorial organizations and bodies | 84 | 0.27 | 1.12 | [0.88,1.43] | 0.95 | [0.74,1.22] | 0.99 | [0.77,1.27] |

[a] Occupation is coded using the International Classification of Occupations, version 1988 (ISCO-88), coded on 1 digit.

[b] Economic activity/industry is coded using the General Classification of Economic Activities (NOGA), based on ISCI third and NACE first revisions

* Univariate model
** Adjusted for age, time period and canton
*** Adjusted for age, time period, canton, civil status, civil status x age and nationality

Statistically significant estimates and p-values<0.05 are shown in bold
