# Supplementary figures and images for: Occupational Factors and Socioeconomic Differences in Breast Cancer Risk and Stage at Diagnosis in Swiss Working Women
Source: Cancers (Basel). 2022 Jul 29;14(15):3713. doi: 10.3390/cancers14153713 (PMC9367372; doi:10.3390/cancers14153713)

## Occupation (2005-2014)

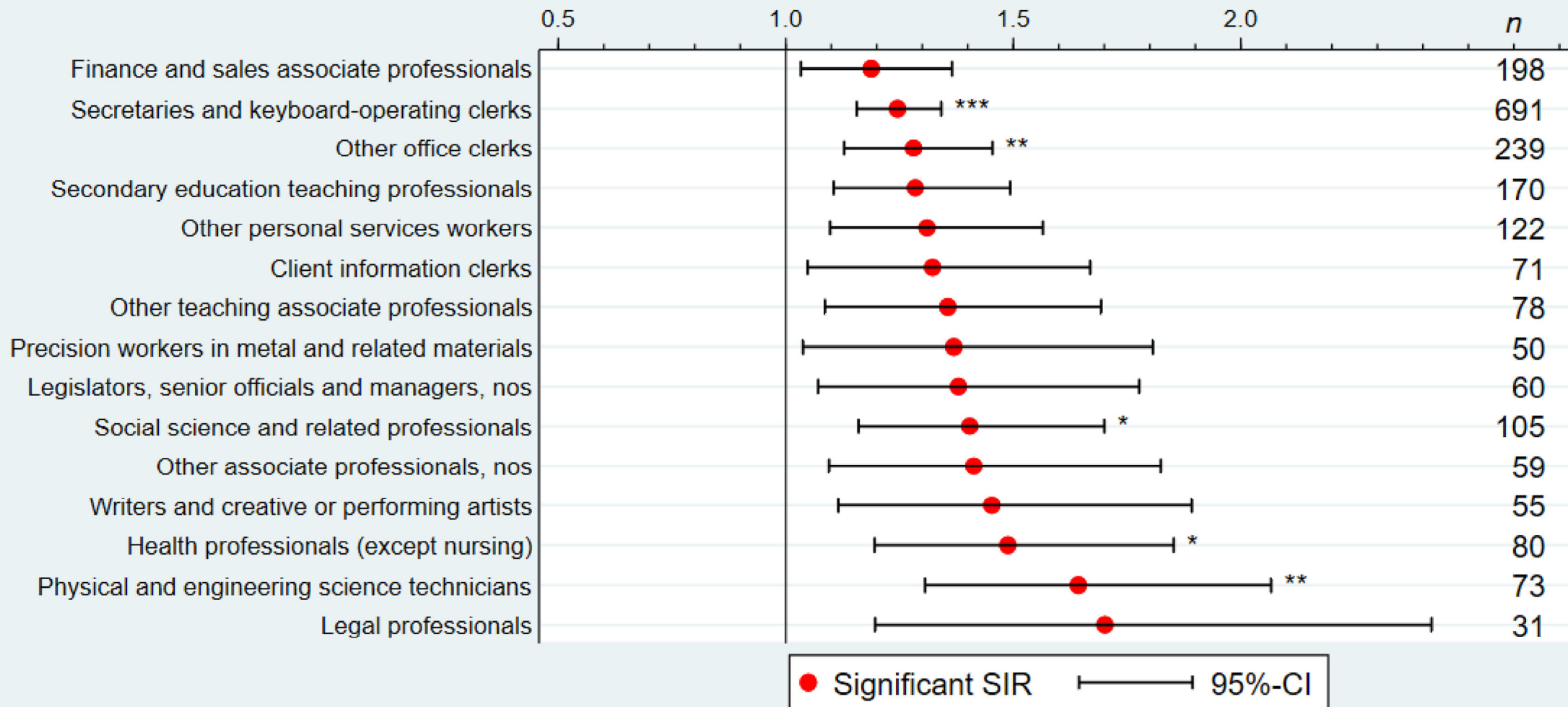

Holm-Bonferroni correction (p-value < 0.001\*\*\*, <0.01\*\*, < 0.05\*)

Supplement: Supplementary file 1 [file cancers-14-03713-s001.zip › FigureS1_SIR_ISCO_88_3d_05_14.pdf]

## Economic activity branch (2005-2014)

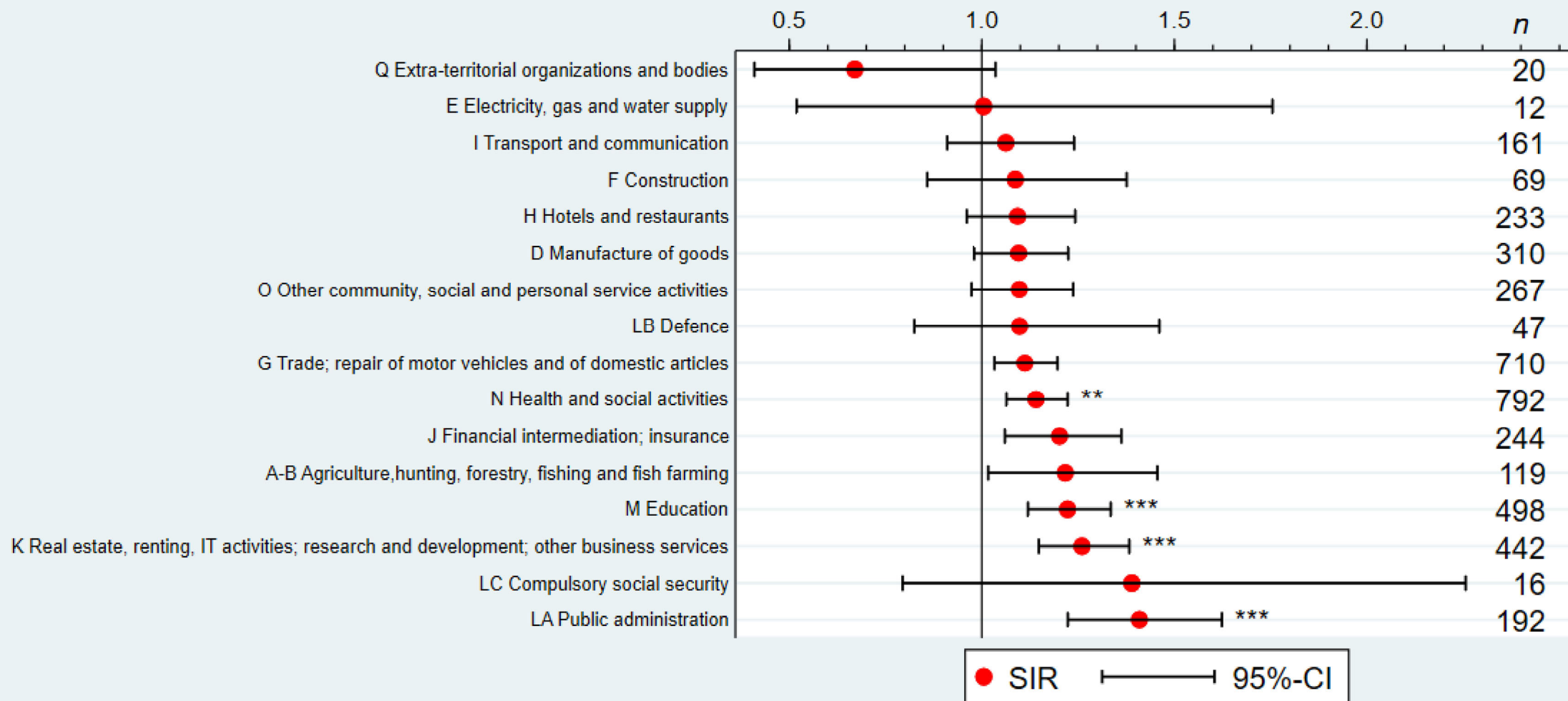

Holm-Bonferroni correction (p-value < 0.001\*\*\*, <0.01\*\*, < 0.05\*)

Supplement: Supplementary file 1 [file cancers-14-03713-s001.zip › FigureS2_SIR_NOGA_95_05_14.pdf]
